# Evaluation of the Conventional Acid-Etching System and the Self-Etching Primer in Bonding Metallic Orthodontic Brackets: An In-Vitro and In-Vivo Study

**DOI:** 10.7759/cureus.72226

**Published:** 2024-10-23

**Authors:** Mohammed Hussin Alomar, Naji Massoud, Mohammad Y Hajeer, Hani Kharma, Doaa Hussain Jomah

**Affiliations:** 1 Department of Orthodontics, The National Dental Center for the Syrian Board and Specialization (NDC) Ministry of Health, Damascus, SYR; 2 Department of Orthodontics, University of Damascus Faculty of Dentistry, Damascus, SYR

**Keywords:** adhesive remnant index, bonding strength, composite, conventional acid etching technique, debonding, failure rate, metallic brackets, premolars, self-etching primer, shear bond strength

## Abstract

Objective: This study aimed to compare the shear bond strength (SBS) of enamel-bonded orthodontic brackets with the conventional acid etching (CAE) system and the self-etching primer (SEP) in vitro and to compare the clinical performance of both systems when used in the treatment of malocclusion patients.

Materials and methods: In the first part of the study, 40 extracted human premolars were randomly divided into two groups containing 20 teeth. The first group (the conventional enamel etching group) employed 37% phosphoric acid before bonding the metallic brackets (0.022-inch slot, MBT prescription, American Orthodontics, Sheboygan, WI, USA). The etching system was Tetric 5th (Ivoclar Vivadent, Schaan, Liechtenstein). The second group used a SEP (Sep Tetric 7th, Ivoclar Vivadent, Schaan, Liechtenstein) to bond the same brackets. In the first part of the study, SBS was evaluated, followed by the adhesive remnant index (ARI) assessment. The second part of the study (i.e., the clinical part) assessed a cohort of 30 patients during a 6-month observation period. The upper 10 teeth (from the second premolar on the right side to the second on the right side) were bonded using the chosen method for each patient in the clinical assessment. That is, 150 teeth in each group were evaluated regarding the failure rate. The ARI was assessed for those teeth that lost their brackets.

Results: The mean SBS was greater in the SEP group compared to the CAE group (17.93 MPa and 16.60 MPa, respectively; P = 0.014). The difference was not statistically significant. Conversely, the failure rate was lower in the CAE group compared to the SEP group, with a failure rate of 6% and 14.7%, respectively. The difference was statistically significant (P = 0.014). However, the ARI showed no statistically significant difference in in-vivo and in-vitro analyses, as most bracket failures were at the adhesive level.

Conclusion: Laboratory results showed no statistical difference in the SBS mean values between the two groups. Clinically, the SEP group showed a greater failure rate than the CAE group, but both failure rates in the two groups were within the clinically acceptable range. The ARI did not show any difference between the two groups in terms of the failure site when the evaluation was conducted in vivo and in vitro, as most of the areas of failure were concentrated in the material itself.

## Introduction

Traditionally in orthodontic bracket bonding, all teeth were acid-etched before bonding the brackets for a successful procedure. The application of acid etching helps remove the smear layer, allowing the brackets to bond directly onto the surface of the etched enamel [1] and reduce the time required for clinical work [2].

Its disadvantages were its high sensitivity to moisture, the loss of the surface of the enamel, and the hypo-mineralization around the bracket base, as the loss of the enamel surface may lead to enamel fractures during bracket removal [3]. The multi-stages of this technique increase its problems. To simplify this technique, self-etching primer (SEP) was introduced in 1988 by Bishara [4], noting that SEP includes phosphoric acid and methacrylate in one compound, etching, and priming at the same time [5]. This solution has shown some advantages over the traditional technique, such as reducing enamel surface loss, preventing contamination, reducing the medical treatment period, and eliminating the rinsing phase [6]. The main components of this substance are methacrylate phosphoric acid esters with a pH of about 1.0. The phosphate group in the phosphoric acid dissolves the calcium and removes it from the hydroxyapatite. Instead of being rinsed away, as with phosphoric acid, the calcium forms a complex with the phosphate group and is incorporated into the hybrid complex after light curing [7], making the etching depth identical to the depth of the primer although the number of adhesion stages is reduced [8]. However, obtaining a low rate of adhesion failure is the most important goal in orthodontics, so it is an obsession to get a clinically sufficient bond strength yet not be too high and prevent damage to the enamel during removing the bracket [9].

Studies related to the research topic are numerous but contradictory: one study reported that the shear bond strength (SBS) of conventional acid etching (CAE) is higher than the SEP with a slight non-significant difference [2]. Another study showed that the SBS of CAE is significantly higher than the SEP [10]. While some researchers reported that the SBS of SEP is higher than that of CAE [11], randomized clinical trials (RCT) indicated that the bond strength of SEP is relatively lower than that of the CAE technique. The majority of studies conducted on SEPs are in-vitro studies. These results cannot be extrapolated to the intraoral environment. Recent clinical studies showed that the self-etch primer can be used effectively in orthodontic bracket bonding. Its clinical performance in bond failure in more than six months differs from the conventional phosphoric acid method and can be mentioned as a suitable alternative [12-13]. Hence, further long-term clinical studies must be conducted before recommending SEPs in routine orthodontic bonding [5].

At the Department of Orthodontics in the National Dental Center for the Syrian Board and Specialization (NDC, Ministry of Health, Damascus, Syria), both types of systems are used depending on the preference of the specialist trainee and his/her clinical supervisor. Thus, this study aimed to compare the SBS of the orthodontic brackets bonded to enamel with the CAE and the SEP techniques in vitro and then assess the failure rate in a cohort of orthodontic patients. An adhesive remnant index (ARI) analysis was also conducted for both parts of this study.

## Materials and methods

Part I: The in-vitro study

Study Design and Settings

This part was conducted within the National Dental Center for the Syrian Board & Specialization (NDC), Damascus, Syria. The Local Research Ethics Committee at the NDC (Reference number: 423 dated 27.5.2023) approved the use of freshly extracted premolars from patients who agreed to allow their teeth to be used in this study.

Sample Size Calculation and Sample Collection

Based on a previous study [14], a sample size estimated at 40 premolars extracted for orthodontic purposes. This part of the study was self-funded. In this part of the study, 40 non-carious premolars were freshly extracted from adults for orthodontic reasons. Premolars were selected based on non-carious, freshly extracted premolars with intact buccal surfaces, with no hypoplastic enamel, fluorosis, restorations, or cracks.

**Figure 1:**
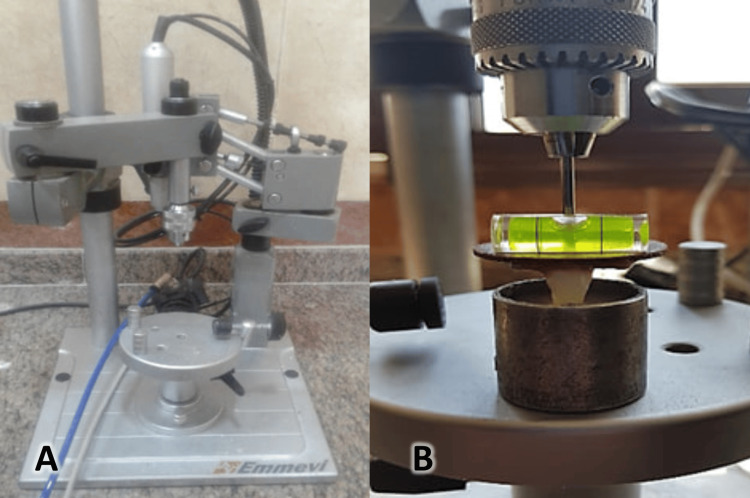
The teeth were cemented in acrylic resin up to the cemento-enamel junction, leaving the crown surface exposed for bonding the brackets with the help of an Emmevi Dental Planner. A) The Emmevi Dental Planner. B) The ejection of the tooth into the acrylic base.

This sample of extracted premolars was kept in distilled water until it was used 48 hours later. The teeth were cemented in acrylic resin up to the cemento-enamel junction (CEJ), leaving the crown surface exposed for bonding the brackets with the help of an Emmevi Dental Planner (Dental Planner, San Maurizio d'Obbalio, Italy) vertical ejection of the tooth to the base (Figure 1). The tooth surface was polished with a slow-speed piece, and the sample was ready to start the bonding process. Teeth with bonded brackets were divided into two groups (each with 20 teeth) according to the type of primer: brackets in the control group were bonded using the CAE group, whereas brackets in the second group were bonded with the SEP method (SEP group).

Stages of Bonding in the CAE Group

Twenty teeth samples were etched with 37% phosphoric acid for 20, rinsed, and air-dried. The etched enamel was covered with a layer of conventional bonding (Tetric 5th, Ivoclar Vivadent, Schaan, Liechtenstein), then dry with airflow, and light-curing was done for 5 seconds, and the metal brackets (0.022Mbt twin, American Orthodontics, Sheboygan, Wis ) that hold the adhesive resin (Reliance Orthodontics Products Inc, Itasca, III, USA) and curing with a high-power light-emitting diode (LED) (Guilin Woodpecker Medical Instrument Co, China) with an intensity of 1200 MW/cm² to ~2500 MW/cm² and 390-480 nm for 5 seconds after confirming the position and adaptability of the bracket and removal of the excess resin.

Stages of Bonding in the SEP Group

Etching and preparation of 20-tooth samples were performed using SEP technology (Tetric 7th, Ivoclar Vivadent, Schaan, Liechtenstein). It was applied to the enamel surface of 20 teeth, rubbed for 3 seconds, and then dried using airflow to remove the excess primer, then the metal brackets were treated for 5 seconds (0.022-inch slot, MBT prescription, American Orthodontics, Sheboygan, Wis, USA) bearing adhesive resin (Reliance Orthodontics Products Inc, Itasca, Ill, USA) and curing with an LED (Guilin Woodpecker Medical Instrument Co, China) with an intensity of 1200 MW/cm² to ~2500 MW/cm² and 390-480 nm for 5 seconds after confirming the position and adaptability of the bracket and removal of excess resin.

Outcome Assessment

**Figure 2:**
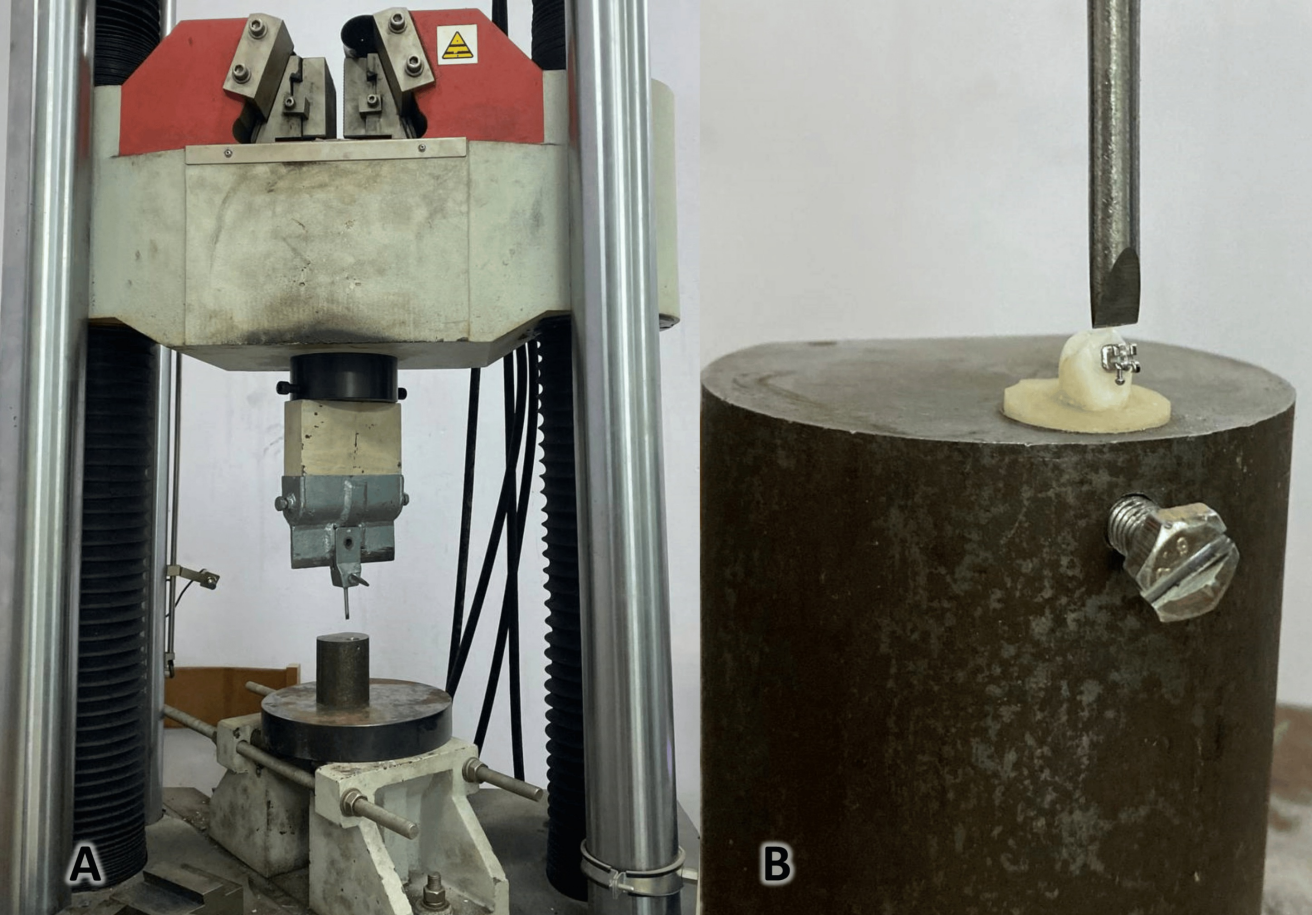
A) Universal Testing Machine (UTM). B) A metal base designed to receive the acrylic base with the tooth, with a cutting blade added to the device.

SBS testing of an in-vitro specimen was performed using a computerized, software-based Universal Testing Machine (Zwick/Ruel, Ulm, Germany). This device was modified by the addition of a cutting blade that was used to apply an occlusal and gingival shear force to the abutment-tooth interface at a tangential velocity of 1 mm/minute, with a metal base designed to contain the acrylic base (Figure 2). The ARI was evaluated according to Artun and Bergland [2] as follows: “Score 0 = no adhesive left on the tooth, score 1 = less than half of the adhesive left on the tooth, score 2 = more than half of the adhesive left on the tooth, score 3 = all of the adhesive left on the tooth.” To obtain the optical images that verify the previous index, a Nikon camera and a lens (Sigma 105 Macro) were used, and the acrylic base was placed at a steady distance of 5 cm and an angle of 90° with the longitudinal axis of the tooth. These images will be computer-processed using AutoCAD-classic software. According to the following equation: Ratio of the remaining adhesive area = remaining adhesive area/total area × 100. The total area of the brackets used was 10.26 mm^2^.

Part II: In the in-vivo study

Study Design and Settings

The second part of this research project was a prospective cohort observational study. This study was conducted at the Orthodontic Department, the NDC, Damascus (Syria), on 30 patients undergoing conventional orthodontic treatment. Ethical approval was obtained from the Local Research Ethics Committee of the NDC (Reference number: 423 dated 27.5.2023). The patient consent form was obtained from the participants upon agreement or from patients’ parents or guardians in the case of teenagers. According to the International Committee of Medical Journal Editors (ICMJE) guidelines, the non-experimental clinical research work protocol does not require registration with clinical trial registries.

Patient Recruitment and the Application of the Clinical Procedures

The size of the sample was determined according to a previous study [14], so the sample included 30 patients who were selected according to the following criteria: patient with permanent occlusion, Class I canine and molar relationships, and crowding less than 5 mm in each jaw to avoid extraction. The age group was from 15 to 20 years. Patients who met the criteria were asked to participate. The principal researcher performed the pre-bonding scaling and polishing procedures. The bonding process was conducted in both groups. The acid-etching technique was applied in the first group on the upper jaw for each patient (i.e., 10 teeth from the second premolar on the right side to the second premolar on the left side), whereas the SEP was used in the second group for the same teeth for each patient. After the bonding was completed, the patients were given the usual instructions, in addition to paying attention to inform the practitioner immediately in the event of any bracket failure.

Outcome Assessment

In the clinical study, the principal researcher (first author) monitored the failure rate and noted the residual material during the first six months of treatment so that the date of failure, location, and tooth type were documented. When the failure occurred, the residual material indicator was documented by taking a picture of the vestibular surface of the tooth with a Nikon optical camera at a distance of 8 cm and an angle of 90° with the longitudinal axis of the tooth.

Statistical analysis for both parts of the study

For the first part of the study, descriptive statistics of the SBS of the two groups were calculated and presented. A t-test was used to determine the statistically significant differences in mean SBS between the two groups. The difference in the ARI score was compared between the two groups using the Mann-Whitney test. For the clinical part of the study, the Chi-square test was used to detect significant differences between the two groups regarding the failure rate. The difference in the ARI score was compared between the two groups using the Mann-Whitney test.

## Results

Part I: The in-vitro study: The shear bond strength and the adhesive remnant index

The mean SBS for the CAE and SEP groups was 16.6 MPa and 17.93 MPa, respectively. As shown in Table 1, the paired t-test revealed no statistically significant difference between the two study groups (P=0.543).

**Table 1:** Descriptive statistics of the shear bond strength (in Megapascal) in the two groups along with the p-value of significance testing. n: number of premolars being examined, SD: Standard deviation *Two-sample t-test.

Group	Mean	SD	Range	T-value	P-value*
Conventional acid etching (n=20)	16.60	5.90	8.3-26.7	0.614	0.543
Self-etching primer (n=20)	17.93	7.74	6.2-38.1		

Regarding the ARI scores, the most frequent scores in the CAE and SEP groups were 2 and 3, respectively (Table 2). The ARI’s scores are almost similar in both groups. According to the Mann-Whitney test result, there was no statistically significant difference between the two groups (P=0.831; Table 3).

**Table 2:** The raw data of the 40 premolars included in the in-vitro study when evaluating the adhesive remnant index. CAE: Conventional acid-etching group; SEP: Self-etching primer group; ARI: Adhesive remnant index

CAE		SEP	
Tooth Number	ARI score	Tooth Number	ARI score
1	1	1	1
2	3	2	2
3	3	3	1
4	2	4	1
5	3	5	1
6	1	6	3
7	1	7	2
8	0	8	2
9	2	9	1
10	0	10	2
11	2	11	3
12	1	12	3
13	2	13	1
14	2	14	1
15	1	15	0
16	1	16	2
17	2	17	2
18	1	18	1
19	2	19	0
20	2	20	2

**Table 3:** Distribution of the ARI scores in the two groups in the laboratory part of the study along with the p-value of significance testing. N: sample size *The results of Mann-Whitney U test.

Group	ARI scores				Z-value	P-value*
	0	1	2	3		
Conventional acid etching (n=20)	2	7	8	3	−0.215	0.830
Self-etching primer (n=20)	2	8	7	3		

Part II: The clinical study

Baseline Sample Characteristics

The study included 30 patients, with 15 patients in each group. The mean age of the CAE group was 18.20 years, whereas that of the SEP group was 17.33 years (Table 4). The difference between the two groups was insignificant (P=0.122). The sex distribution in both groups was similar, with three males and 12 females in each group.

**Table 4:** Baseline sample characteristics of the included patients in this study regarding age and gender. *Two-sample t-test ​​​​​​​^†^Two-proportion test. CAE: Conventional acid-etching group; SEP: Self-etching primer group; SD: Standard deviation; M: F distribution: Male to female distribution; n: number of patients included in this study

Group	Age (Mean ± SD)	M: F distribution
CAE (n=15)	18.20 ± 1.42	3: 12
SEP (n=15)	17.33 ± 1.54	3: 12
P-value	0.122*	≈1.00^†^

Failure Rate and the Adhesive Remnant Index Evaluation

In the CAE group, the number of failed brackets was 9 (6 %) out of the 150 brackets bonded in this group (i.e., ten brackets for each patient), whereas, in the SEP group, it was 22 brackets (14.7 %) out of 150 brackets; the observed difference was statistically significant between the two groups (P=0.014; Chi-square test; Table 5). Regarding the ARI score when evaluating the teeth with failed brackets, the most frequent scores in the CAE and SEP groups were 2 and 0, respectively (Table 6). The difference between the two groups in the observed pattern distribution was not statistically significant.

**Table 5:** Distribution of the success/failure rates in the clinical part of the study along with the P-value of significance testing. *Result of Chi-square test; n: number of teeth under evaluation (15 patients in each group with 10 teeth bracketed for each patient).

Group	Success	Failure	Chi-square value	P-value*
Conventional acid etching (n=150)	141 (94%)	9 (6%)	6.08	0.014
Self-etching primer (n=150)	128 (85.3%)	22 (14.7%)		

**Table 6:** Distribution of the ARI scores of the teeth whose brackets failed in the two groups along with the p-value of significance testing. n: number of teeth with failed brackets whose surfaces were examined for the ARI analysis; ARI: adhesive remnant index *Results of Mann-Whitney U test.

Group	ARI scores				Z-test value	P-value*
	0	1	2	3		
Conventional acid etching (n=9)	3	2	4	0	0.925	0.355
Self-etching primer group (n=22)	10	8	2	2		

## Discussion

Several previous studies have shown that the SBS of CAE was significantly greater or similar to that of SEP. In the current study, statistical results showed no significant differences in the mean values of SBS of the CAE and SEP, with mean values of 16.6 and 17.9 MPa, respectively. Both values were greater than the minimum clinically acceptable values of 6-8 MPa [6], and our results are consistent with those of Yadala et al. [6]. This consistency may be explained by the matching etching obtained with the two systems, except that the etching depth is less with the self-etching system [15]. In contrast, Zope et al.’s study found that the average SBS values for the conventional brackets were greater than those of the SEPs when used with metal brackets [1]. Asgari et al. indicated that the average SBS values for the self-etching group are higher than those for the conventional group [16]. This discrepancy in the results may be due to several factors, including the quality of the force applied, the adhesive material, the study model, tooth preparation, and storage time.

Yadala et al. reported the conventional technique relies on its bond strength when applying phosphoric acid, which creates an irregular enamel surface by dissolving minerals between the enamel prisms, facilitating the bonding of the brackets [6]. The high bond strength using self-etch primers has been attributed to nano-retentive interlocking between the enamel crystallites and the resin, in addition to chemical bonding [14]. The conventional bond washing stage may cause a lot of unsupported enamel to be lost, reducing the bonding areas, whereas if a self-etching bond is applied, the enamel is left intact and undisturbed, which provides more enamel for bonding and reduces enamel loss [7]. The self-etched bond etches and works as a primer at the same time, and this makes the resin penetrate the entire etched surface, preventing the possibility of a completely hypomineralized surface that is not penetrated by the resin [17].

ARI showed a failure pattern within the adhesive interface, which is more desirable because it is not excessively strong, which causes a crack in the enamel. It is also easy to clean and does not damage the enamel while removing residue, which coincides with [1].

Our study demonstrated inconsistency between the in-vivo and in-vitro findings, with the SEP showing a higher average SBS than the CAE in vitro, while the clinical adhesion failure rate was higher with the SEP than CAE with an average rate of 14.7% and 6%, respectively. However, the failure rate in both groups remained within the clinically acceptable range of 2.7 - 23% [17]. Our study is almost similar to Elekdag-Turk et al., which showed a significant difference between the bond failure rates of the SEP and CAE group were 4.7% and 1.7%, respectively [18]. This failure rate can be attributed to the fact that conventional etching makes the enamel surface rougher and thus makes the enamel more stable, increasing the bond strength, which may be higher than clinically desirable [19]. As for the SEP group, Gandhi et al. reported the etching depth was less than that of the CAE group due to the lower acidity in SEP [20].

In addition, Littlewood et al. reported that although the conventional system is hydrophobic, the SEP contains hydrophilic monomers and solvents, which makes it less sensitive to moisture. However, these monomers increase water absorption, which may weaken the adhesive interface and lead to bracket debonding [21]. This difference between the in-vitro and in-vivo studies can be explained by the difference in the oral environment from the laboratory conditions, which were on dry enamel, in addition to the difference in the forces applied clinically, such as tension, shear, and chewing forces, while in the laboratory, they were limited to shear forces.

On the contrary [15-20], no difference was shown between the two agents, but in some studies, the self-etch primer showed a lower failure rate than the conventional technique. This contradicts the results of our research, which can be explained by the fact that some of these studies did not follow the manufacturer's instructions. The manufacturer works to increase the rubbing time, which may lead to losing the most important advantage of self-etch primer, which is reducing the clinical work time, in addition to factors related to the patient’s environment, eating habits, masticatory forces, and facial pattern. The clinical residual index showed no difference in failure sites, consistent with the study [15]. However, due to the various factors that cause brackets to fail, failure at the material/bracket level can be assured to be safer because it does not cause cracks in the enamel, while failure at the material/enamel level is desirable for easy cleaning [6]. Finally, based on the results of the study that showed the failure rate of dental systems within the clinically acceptable range, self-etch can be used as an alternative material in areas that are difficult to isolate, as it is a hydrophilic material, as long as the tooth surface is well prepared and plaque is removed.

Limitations of the current work

There are a few limitations to the current study. First, the clinical comparison was limited to patients with Class I malocclusion according to Angle’s classification, with crowding of less than 5 mm. The failure rate may arise with patients with more complex scenarios. Second, the comparison was made during the first six months of treatment without considering the diameter and stiffness of the placed archwires, which may have affected the intensity of the applied forces. Third, the sample included patients from both sexes, with a predominance of female patients. The sex-related differences in the chewing forces may have played a role in the failure rates of the bonded brackets. In addition, the clinical study evaluated the bonded brackets on the upper jaw only. Finally, there is a need for more long-term studies that include patients with different types of malocclusion and treatment modalities.

## Conclusions

Laboratory results showed no statistical difference in the SBS mean values between the two groups. Clinically, the SEP group showed a greater failure rate than the CAE group, but both failure rates in the two groups were within the clinically acceptable range. The ARI did not show any difference between the two groups regarding the failure site when the evaluation was conducted in vivo and in vitro, as most of the areas of failure were concentrated in the material itself.
